# Адипсический несахарный диабет после транссфеноидального удаления стебельно-интравентрикулярной краниофарингиомы

**DOI:** 10.14341/probl13126

**Published:** 2022-06-06

**Authors:** Л. И. Астафьева, И. Н. Бадмаева, Ю. Г. Сиднева, И. С. Клочкова, Д. В. Фомичев, И. В. Чернов, П. Л. Калинин

**Affiliations:** Национальный медицинский исследовательский центр нейрохирургии им. академика Н.Н. Бурденко; Национальный медицинский исследовательский центр нейрохирургии им. академика Н.Н. Бурденко; Национальный медицинский исследовательский центр нейрохирургии им. академика Н.Н. Бурденко; Научно-исследовательский институт неотложной детской хирургии и травматологии Департамента здравоохранения города Москвы; Национальный медицинский исследовательский центр нейрохирургии им. академика Н.Н. Бурденко; Национальный медицинский исследовательский центр нейрохирургии им. академика Н.Н. Бурденко; Национальный медицинский исследовательский центр нейрохирургии им. академика Н.Н. Бурденко; Национальный медицинский исследовательский центр нейрохирургии им. академика Н.Н. Бурденко

**Keywords:** центральный несахарный диабет, адипсический несахарный диабет, гипернатриемия, жажда, адипсия, краниофарингиома, десмопрессин

## Abstract

Представленный клинический случай демонстрирует редкую патологию диэнцефальной области — адипсический несахарный диабет (АНД) с развитием тяжелой гипернатриемии у женщины 58 лет после эндоскопического трансназального транссфеноидального удаления стебельно-интравентрикулярной краниофарингиомы. АНД у пациентки диагностирован на основании гипернатриемии (150–155 ммоль/л), полиурии (до 4 л в сутки) и отсутствия чувства жажды. На фоне терапии десмопрессином и адекватного восполнения жидкости в послеоперационном периоде отмечена нормализация водно-электролитного баланса. Однако самостоятельное прекращение пациенткой терапии десмопрессином после выписки из стационара и отсутствие адекватного потребления жидкости на фоне полиурии привели к выраженной гипернатриемии (155–160 ммоль/л) и грубым психическим нарушениям.

Пациенты с АДН нуждаются в строгом мониторинге клинического состояния и параметров водно-электролитного баланса, в назначении фиксированных доз десмопрессина, а также в адекватном восполнении жидкости.

## Abstract (en)

Presented case demonstrates a rare diencephalic pathology — adipsic diabetes insipidus (ADI) with severe hypernatremia in a 58-year-old woman after ttranssphenoidal removal of stalk intraventricular craniopharyngioma. ADI was diagnosed because of hypernatremia (150–155 mmol/L), polyuria (up to 4 liters per day) and absence of thirst. Normalization of water-electrolyte balance occurred on the background of desmopressin therapy and sufficient hydration in postoperative period. After release from the hospital, the patient independently stopped desmopressin therapy and did not consume an adequate amount of fluid of the background of polyuria. This led to severe hypernatremia (155–160 mmol/L) and rough mental disorders.

Patients with ADI need closely monitoring of medical condition and water-electrolyte parameters, appointment of fixed doses of desmopressin and adequate hydration.

## АКТУАЛЬНОСТЬ

Краниофарингиома (КФ) — редкая эмбриональная опухоль с низкой гистологической степенью (ВОЗ), развивающаяся из остатков краниофарингеального протока в хиазмально-селлярной области. Частота встречаемости этих опухолей составляет 1,2–4,6% [1]. Общая выживаемость пациентов с КФ высока (87–95%), однако качество жизни часто ухудшается из-за последствий, обусловленных анатомической близостью к гипоталамо-гипофизарной области и зрительному перекресту [2].

Несахарный диабет (НД) — это наиболее распространенное послеоперационное нарушение после хирургического лечения КФ, частота его развития составляет 60–96% [2–4].

У пациентов с КФ и НД более высокий показатель смертности, чем у пациентов без НД [5]. Вероятно, это ассоциировано с более частым поражением гипоталамических структур, а также тяжелой диснатриемией. В редких случаях развивается адипсический НД. Аномалии жажды могут привести к опасным для жизни электролитным и метаболическим последствиям, что увеличивают смертность у пациентов с КФ [2][4].

Представленный клинический случай демонстрирует адипсический НД с развитием тяжелой гипернатриемии и грубых психических нарушений после эндоскопического трансназального транссфеноидального удаления стебельно-интравентрикулярной КФ.

## ОПИСАНИЕ СЛУЧАЯ

Пациентка З., 58 лет, 09.11.2021 поступила в ФГАУ «НМИЦ нейрохирургии им. акад. Н.Н. Бурденко» МЗ РФ с жалобами на ухудшение зрения.

Из анамнеза: указанные жалобы беспокоили в течение года. В 2015 г. пациентке проведена левосторонняя нефрэктомия по поводу светлоклеточного рака почки, Т1аN0M0. Также известно, что около 10 лет назад диагностирован сахарный диабет 2 типа. За 6 мес до поступления перенесла инфекцию COVID-19.

Гинекологический анамнез: 2 беременности, закончившиеся срочными родами путем кесарева сечения. Постменопауза с 53 лет.

При поступлении состояние удовлетворительное. Пациентка не предъявляла жалоб на жажду, учащенное мочеиспускание. Диурез не учитывался. Рост 167 см. Вес 95 кг. ИМТ 34,06 кг/м2. Кожные покровы чистые, физиологической окраски. Подкожно-жировая клетчатка развита избыточно, распределение преимущественно равномерное. АД 130/80 мм рт.cт., ЧСС — 78 в минуту. Неврологический статус и психическое состояние без особенностей.

При нейроофтальмологическом обследовании выявлены признаки воздействия на зрительный путь на основании головного мозга. Vis OD=1,0, Vis OS=1,0, сужение поля зрения в височной половине OD, концентрическое сужение поля зрения ОS.

В гормональном анализе крови гипопитуитарных нарушений выявлено не было (табл. 1). В биохимическом анализе крови отмечена умеренная гипернатриемия 148 ммоль/л (референсный интервал 136–145). В клиническом анализе мочи удельная плотность составила 1013 г/л. Показатели углеводного обмена компенсированы на фоне приема пероральных сахароснижающих препаратов (гликлазид 60 мг, линаглиптин 5 мг и метформин 1000 мг), гликированный гемоглобин 6,7%.

**Table:** Таблица 1. Лабораторные показатели пациентаTable 1. Patient's laboratory parameters Примечание. ТТГ — тиреотропный гормон; Т4 — тироксин; Т3 — трийодтиронин; ЛГ — лютеинизирующий гормон; ФСГ — фолликулостимулирующий гормон; ИФР-1 — инсулиноподобный фактор роста 1.

Показатель	Результат до операции	Результат после операции	Результат на 28 сутки после операции на фоне отмены гормональной терапии	Референсный интервал	Единицы измерения
ТТГ	1,4	0,3	0,2	0,35–4,94	мМЕ/мл
Т4 свободный	11,8	8,5	9,7	9,0–19,0	пмоль/л
Т3 свободный	3,8	3,1	2,92	3,5–6,5	пмоль/л
Кортизол	256	27	31	101–536	нмоль/л
Пролактин	546	450	352	110–562	мкМЕ/мл
ЛГ	-	0.1	0,1	5,16–61,99	Ед/л
ФСГ	-	0,2	0,3	26,72–133,41	Ед/л
Эстрадиол	-	28	25	36–528	пмоль/л
ИФР-1	86	78	71,3	81–225	нг/мл

При проведении МРТ головного мозга выявлена солидно-кистозная супраселлярная КФ с интравентрикулярным распространением, вовлечением стебля гипофиза (рис. 1).

**Figure:**
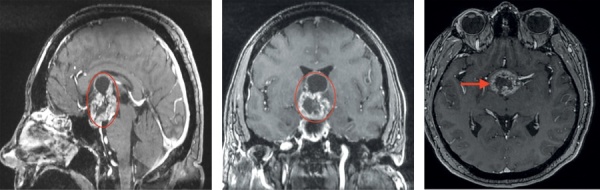
Рисунок 1. МРТ пациентки З. до операции. Визуализируется солидно-кистозная супраселлярная краниофарингиома с интравентрикулярным распространением, вовлечением стебля гипофиза (обозначена и указана стрелкой).Figure 1. MRI of patient Z. before surgery. A solid cystic suprasellar craniopharyngioma with intraventricular extension involving the pituitary stalk is visualized (marked and indicated by an arrow).

10.11.2021 проведено эндоскопическое трансназальное удаление стебельно-интравентрикулярной КФ. В ходе операции обнаружен солидный и кистозный компоненты опухоли, происходящей из стебля гипофиза. После вынужденного пересечения стебля гипофиза и мобилизации опухоли она была удалена крупными фрагментами, включая практически всю капсулу. Остались небольшие фрагменты капсулы, которые были плотно спаяны с дном 3 желудочка и хиазмой зрительных нервов. После удаления основного объема опухоли визуализирован III желудочек, межталамическое сращение.

По данным послеоперационного КТ-исследования осложнений не выявлено. Гистологическое исследование подтвердило адамантиномоподобную КФ, WHO Grade I, с выраженной глиальной капсулой.

В 1-е сутки после операции отмечалось повышение уровня натрия до 155 ммоль/л (референсный интервал 136–145 ммоль/л). Пациентка предъявляла жалобы на головную боль, отмечался эпизод психомоторного возбуждения, потребовавший дополнительного назначения седатирующей терапии. Получала дексаметазон, антипсихотические и противоэпилептические препараты (кветиапин, аминомасляную кислоту, вальпроат натрия). При контроле гормонального анализа на фоне отмены дексаметазона был диагностирован пангипопитуитаризм: вторичный гипотиреоз, вторичная надпочечниковая недостаточность, вторичный гипогонадизм, дефицит соматотропного гормона (табл. 1). Была назначена терапия гидрокортизоном в дозе 30 мгв сутки и левотироксином натрия 100 мкг в сутки. В качестве сахароснижающей терапии применялся инсулин короткого действия, впоследствии была переведена на базис-болюсную инсулинотерапию, на этом фоне гликемия 7–12 ммоль/л. На 2-е сутки после операции по данным учета диуреза объем выпитой жидкости составил 1000 мл, выделенной — 2300 мл. Уровень натрия 150–155 ммоль/л, осмоляльность крови 323 мОсм/л (референсный интервал 280–295 мОсм/л). Несмотря на это, произвольной потребности в приеме жидкости пациентка не испытывала.

В связи с отсутствием у пациентки полидипсии при увеличенном объеме выделенной жидкости, стойкой гипернатриемии диагностирован адипсический НД. Назначен десмопрессин 0,1 мг 2 раза в сутки, с дополнительным приемом при повышении темпов диуреза при соблюдении питьевого режима (не менее 2000–2500 мл жидкости в сутки). На этом фоне показатели натрия стабилизировались в пределах 140–143 ммоль/л, объем выпитой жидкости соответствовал выделенному. На 5-е сутки нафоне полиурии около 4000 мл в сутки вновь отмечена гипернатриемия до 150 ммоль/л. При дополнительном приеме десмопрессина показатели нормализовались. Динамика уровня натрия в крови в периоперационном периоде отражена на рисунке 2.

**Figure:**
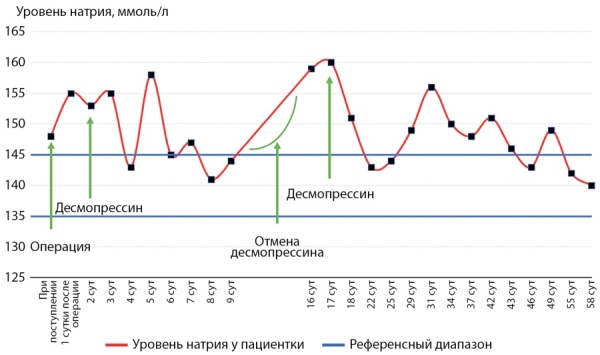
Рисунок 2. Динамика уровня натрия у пациентки З.Figure 2. Dynamics of sodium level in patient Z.

На 8-е сутки после операции пациентка была выписана в удовлетворительном состоянии без явного психоневрологического дефицита. Уровень натрия при выписке 144 ммоль/л. В анализах крови значимых патологических изменений не выявлено. Даны рекомендации по приему гормональной терапии (гидрокортизон, левотироксин натрия, десмопрессин), соблюдению питьевого режима с потреблением не менее 2–2,5 л жидкости в стуки. Также была скорректирована сахароснижающая терапия.

На 16-е сутки после операции (8-е сутки выписки из стационара) пациентка экстренно госпитализирована в отделение в состоянии глубокого оглушения. АД 130/80 мм рт.ст. Со слов родственников, в течение последних 4 дней больная прекратила прием гормональной терапии, не вела учет выпитой и выделенной жидкости. Развились выраженные психические нарушения, когда стала «заговариваться», была грубо дезориентирована — не понимала, где находится, что с ней происходит, не узнавала родственников.

При поступлении в биохимическом анализе крови уровень натрия 155–160 ммоль/л в течение дня, гипергликемия 12–19 ммоль/л. В отделении наблюдалось нарастание психомоторного возбуждения с галлюцинаторно-бредовой симптоматикой. Пациентка стала беспокойной, дезориентированной, отвечала невпопад, разговаривала сама с собой. Стремилась уйти, собирала вещи, разговаривала с «мнимыми» людьми, пыталась «спастись», отмахивалась от кого-то, отрывочно высказывала, что рядом в палате «животные и насекомые». Данные гормонального анализа крови на фоне отмены гидрокортизона и левотироксина приведены в таблице 1.

По данным МРТ-исследования признаков интракраниальных осложнений не выявлено, фрагменты капсулы опухоли в области III желудочка (рис. 3).

**Figure:**
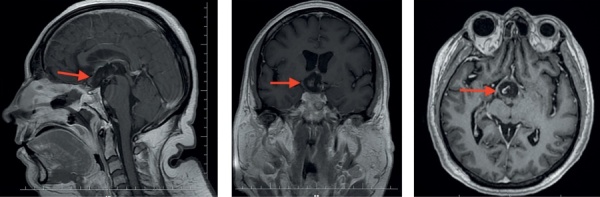
Рисунок 3. МРТ пациентки З. после операции. На фоне послеоперационных изменений визуализируются фрагменты капсулы опухоли в области III желудочка (указаны стрелкой).Figure 3. MRI of patient Z. after surgery. Against the  background of postoperative changes, fragments of the tumor capsule are visualized in the region of the third ventricle (indicated by an arrow).

Возобновлена гормональная терапия: преднизолон в дозе 30 мг, левотироксин в дозе 100 мкг, десмопрессин 0,1 мг 2–3 раза в день под контролем диуреза и уровня натрия. В связи с гипергликемией проводилась постоянная внутривенная инфузия инсулина с дальнейшей нормализацией показателей в пределах целевых значений. На фоне проводимой терапии десмопрессином постепенно достигнуто снижение уровня натрия (143–144 ммоль/л), суточный диурез 2500–3500 мл, сохранялись психические нарушения в эмоционально-личностной и мотивационной сфере, мнестические расстройства. Ввиду снижения произвольной потребности в приеме жидкости пациентка самостоятельно выпивала не более 500–600 мл в сутки, запивая пищу. Для достижения нормального водного баланса требовались уговоры медицинского персонала выпивать не менее 2500 мл жидкости в сутки.

Выписана с рекомендациями по приему преднизолона 7,5 мг, левотироксина 100 мкг и фиксированных доз десмопрессина 60 мкг (0,1 мг) 3 раза в сутки под постоянным контролем количества выпитой и выделенной жидкости со стороны пациенткии ее родственников, динамическим исследованием электролитов крови.

## ОБСУЖДЕНИЕ

Адипсический НД (АНД) является редким и потенциально опасным для жизни заболеванием. Он характеризуется центральным НД вследствие дефицита вазопрессина (синоним: антидиуретический гормон, АДГ) и отсутствием нормальной реакции на жажду при гиперосмолярности. В литературе случаи НД с адипсией или гиподипсией встречаются редко. По данным работы Y. Eisenberg, А. Lawrence (2016 г.), всего в литературе описано около 100 случаев адипсически-гиподипсического НД [6–10].

Жажда и секреция АДГ являются важнейшими процессами поддержания водно-электролитного баланса. Возникновение жажды является защитным механизмом, что позволяет проводить адекватную регидратацию в ответ на полиурию, тем самым поддерживая нормальную концентрацию натрия. Основным фактором, влияющим на секрецию вазопрессина и реакцию на жажду, является изменение осмоляльности плазмы крови; нормальная осмоляльность варьирует в пределах 285–295 мОсм/кг. Повышение осмоляльности стимулирует жажду и высвобождение вазопрессина. Как известно, тела нейронов, продуцирующих АДГ, расположены в супраоптических и паравентрикулярных ядрах гипоталамуса, аксоны которых заканчиваются в задней доле гипофиза. Осморецепторы жажды также расположены в передних отделах гипоталамуса и терминальной пластинки. Возникновение послеоперационного НД связывают с интраоперационным повреждением гипофиза и его стебля, что приводит к прерыванию транспорта АДГ из гипоталамуса, нарушению его высвобождения из задней доли гипофиза, либо при повреждении аксонов АДГ-секретирующих нейронов обусловливает ретроградное (восходящее) повреждение ядер гипоталамуса, секретирующих АДГ. Причиной нарушения возникновения чувства жажды считают повреждение осморецепторной области гипоталамуса [9–11].

Потеря чувства жажды затрудняет диагностику и увеличивает риск обезвоживания и тяжелой гипернатриемии (содержание натрия в сыворотке более 150 ммоль/л). Как было сказано выше, пациенты с АНД подвержены высокому риску тяжелой гипернатриемии. Н. Arima и соавт. сообщили об относительном риске гипернатриемии в сыворотке крови у пациентов с АНД в сравнении с пациентами с НД и сохраненной жаждой (25% против 0,4% от измеренных значений натрия) [12].

Гипернатриемия и дегидратация могут проявляться выраженной цефалгией, гипертермией, тошнотой, рвотой, судорогами, психомоторным возбуждением, нарушением сознания. Диагноз адипсии/гиподипсии может быть установлен, когда пациент с гипернатриемией или гиперосмоляльностью отрицает жажду и/или не пьет самопроизвольно или даже проявляет отвращение к жидкости. НД у таких пациентов, как правило, постоянный, но со временем может появиться жажда, в ряде случаев заболевание разрешается в течение года после операции. Предполагается, что это связано с тем, что осморецепторы, контролирующие чувство жажды, в отличие от клеток, секретирующих АДГ, сохраняют некоторую способность к восстановлению. Чаще такое состояние встречается у пациентов с КФ, причем как до оперативного лечения, так и после него [4][13–16].

Тактика ведения пациентов с АНД аналогична алгоритму лечения пациентов с центральным НД различного генеза: нормализация диуреза и уровня натрия. Стойкая гипернатриемия диктует необходимость более длительного наблюдения в условиях стационара с целью своевременной коррекции терапии, в том числе и у бессимптомных пациентов [12].

Учитывая редкость встречаемости НД с адипсией, в настоящее время крупных клинических исследований по оценке эффективности различных схем лечения не проводилось. По имеющимся данным литературы, чаще применяются фиксированные дозы десмопрессина для достижения суточного диуреза от 1,5 до 2 л. Также нормализация уровня натрия кроме лекарственной терапии достигается путем достаточного потребления воды, чтобы избежать гипернатриемии при обезвоживании. Однако избыточное потребление жидкости, когда секреция АДГ не нарушена или во время лечения аналогами АДГ, может способствовать водной интоксикации и гипонатриемии. Обязателен контроль веса для оценки потерь жидкости [10][13][16–18].

## ЗАКЛЮЧЕНИЕ

Представленный клинический случай демонстрирует редкую патологию диэнцефальной области — адипсический НД с развитием тяжелой гипернатриемии у пациентки со стебельно-интравентрикулярной КФ. Его развитие, вероятно, обусловлено опухолевым поражением гипоталамического «центра жажды» до проведения хирургического лечения, а интраоперационное пересечение стебля гипофиза усугубило течение НД.

Таким образом, всем пациентам с КФ независимо от жалоб обязательно проведение до и после оперативного вмешательства гормонального исследования, учета количества выпитой и выделенной жидкости, контроля уровня натрия, массы тела. Крайне важно исследование осмоляльности крови и мочи у пациентов с патологией диэнцефальной области. Ранняя диагностика нарушений и своевременное назначение препаратов десмопрессина в послеоперационном периоде улучшает прогноз послеоперационной реабилитации и снижает риски развития осложнений и смертности у таких пациентов.

## ДОПОЛНИТЕЛЬНАЯ ИНФОРМАЦИЯ

Источник финансирования. Статья подготовлена на личные средства авторского коллектива.

Согласие пациента. Пациент добровольно подписал информированное согласие на публикацию в журнале Проблемы эндокринологии персональной медицинской информации в обезличенной форме.

Конфликт интересов. Авторы декларируют отсутствие явных и потенциальных конфликтов интересов, связанных с публикацией настоящей статьи.

Участие авторов. Астафьева Л.И., Бадмаева И.Н., Сиднева Ю.Г. — существенный вклад в концепцию или дизайн исследования, в получение, анализ данных или интерпретацию результатов; Астафьева Л.И., Бадмаева И.Н., Сиднева Ю.Г., Клочкова И.С., Фомичев Д.В., Чернов И.В., Калинин П.Л. — написание статьи или внесение в рукопись существенной (важной) правки с целью повышения научной ценности статьи; Калинин П.Л. — одобрение финальной версии рукописи.

Все авторы одобрили финальную версию статьи перед публикацией, выразили согласие нести ответственность за все аспекты работы, подразумевающую надлежащее изучение и решение вопросов, связанных с точностью или добросовестностью любой части работы

Согласие пациента. Пациент добровольно подписал информированное согласие на публикацию персональной медицинской информации в обезличенной форме.
